# Virtual Network Embedding Based on Topology Potential

**DOI:** 10.3390/e20120941

**Published:** 2018-12-07

**Authors:** Xinbo Liu, Buhong Wang, Zhixian Yang

**Affiliations:** Information and Navigation college, Air Force Engineering University, Xi’an 710077, Shaanxi, China

**Keywords:** network virtualization, virtual network embedding, topology potential, topology potential entropy

## Abstract

To improve the low acceptance ratio and revenue to cost ratio caused by the poor match between the virtual nodes and the physical nodes in the existing virtual network embedding (VNE) algorithms, we established a multi-objective optimization integer linear programming model for the VNE problem, and proposed a novel two-stage virtual network embedding algorithm based on topology potential (VNE-TP). In the node embedding stage, the field theory once used for data clustering was introduced and a node embedding function designed to find the optimal physical node. In the link embedding stage, both the available bandwidth and hops of the candidate paths were considered, and a path embedding function designed to find the optimal path. Extensive simulation results show that the proposed algorithm outperforms other existing algorithms in terms of acceptance ratio and revenue to cost ratio.

## 1. Introduction

In recent years, network virtualization technology has mainly been of academic interest. It is not only a key technology to solve the ossification problem of the Internet [1,2,3], but also a core technology in cloud computing [4,5,6]. In the future Internet, through network virtualization, the current Internet service providers will be decoupled into two independent entities: infrastructure providers and service providers. Infrastructure providers are responsible for deploying and managing the physical network, while service providers are responsible for assembling, installing, and managing virtual networks and offering services through them. Such an Internet paradigm makes it possible to introduce new network architectures, protocols, and services without changing the existing network, effectively supporting the innovation of network technologies and overcoming the Internet ossification problem [7]. In the cloud computing environment, through network virtualization, users can be provided with flexible, scalable, and self-service resource leasing services, which can effectively promote the development of cloud computing.

As a resource allocation problem in the process of network virtualization, virtual network embedding (VNE) is one of the major challenges in network virtualization [8]. Because VNE is limited by node resource constraints, link resource constraints, access control conditions, and random arriving of virtual network requests (VNRs), the solution to the VNE problem is extremely complex. It has been proved that the VNE problem is NP-hard [9]. Although the VNE problem can be solved by exact solutions [10,11], when the instances of the problem are large, it is difficult to solve it in a limited time.

In order to make a tradeoff between the solution quality and the execution time, most scholars decompose the VNE problem into two stages: node embedding and link embedding, and use heuristic-based algorithms to solve it. In these two-stage VNE algorithms, different kinds of heuristic approaches are usually used in the node embedding stage, which are also the main contributions of these algorithms; and the shortest path, k-shortest path or multiple commodity flow algorithm is usually adopted in the link embedding stage.

In the node embedding stage, most of the early scholars [12,13,14] only adopted simple greedy strategies to embed virtual nodes onto the physical nodes with the highest local resource. Since these algorithms only consider the resources of the nodes themselves, the topology attributes of adjacent nodes are neglected, which results in poor coordination of node embedding and link embedding, thus decreasing the performances of the VNE algorithms.

In order to better solve the VNE problem, some scholars have recently proposed several coordinated two-stage VNE algorithms, which consider link embedding when solving node embedding. Zhao et al. [15] take the neighborhood relationship between nodes into consideration in the node embedding stage, which can shorten the link embedding distance and improve the acceptance ratio and revenue to cost ratio. Ding et al. [16] borrow the betweenness centrality in graph theory to rank the nodes in virtual networks, and sort the nodes of the physical network according to the correlation properties between the former selected and unselected nodes. In this way, node embedding and link embedding are well coordinated. Similar to literature of ref. [16], Zhang et al. [17] propose a novel node importance evaluating method with the purpose of choosing the most important physical node so as to facilitate the subsequent link embedding. The main contributions of their work are the definitions of the node degree and clustering coefficient information. Inspired by the successful PageRank algorithm used by Google’s search engine, the Markov Random Walk Model [18] and the Markov Reward Model [19] are introduced to measure the importance of nodes in a network. In their algorithms, each node is ranked based not only on its local resources but also on the resources of the nodes from which it can reach. In this way, the information about the links, necessary for the link embedding stage, is also considered during the node embedding stage. In recent years, multi-criteria decision analysis methods such as TOPSIS [20] and ELECTRE [21] have been introduced into VNE algorithms. In these algorithms, first, the authors define several attributes of nodes from both local and global perspectives, and then use a multi-criteria decision analysis method to rank the nodes in the network. In this way, the most appropriate physical nodes will be selected to host the virtual nodes, which will facilitate subsequent link embedding. Though, in all of the above methods, the network topology structure is used to evaluate the resource capabilities of nodes, which makes the results of node ranking more reasonable and improves the embedding performance of VNRs, in our opinion, some special topological attributes of nodes can be further explored and utilized to improve the embedding performance of VNRs.

Different from the above research, in this paper, we consider the topological importance of nodes in the network from a new perspective. In physics, the field represents the distribution of a physical quantity in space, which can be used to describe the interaction between non-contact objects. Gan et al. [22] introduced field theory into abstract data space. In their paper, the importance of data objects can be clarified by describing the virtual interactions among data objects. Inspired by the above theory, we introduced the field theory into the VNE process and used the topology potential to measure the topology importance of nodes.

The main innovations and contributions of this paper can be summarized as follows:

(1) To describe the topology importance of a node in a network from a global perspective, a new topological attribute named “node topology potential” is defined, which can be utilized to further improve the embedding performance of VNRs.
(2) A novel virtual network embedding algorithm based on topology potential (VNE-TP) is proposed. The VNE-TP algorithm considers the importance of the nodes in terms of topology and resources in the node embedding stage, and considers the available bandwidth and path length in the link embedding stage.
(3) The simulation results are provided to validate the performance of the VNE-TP algorithm. It can be demonstrated that the VNE-TP algorithm outperforms the existing algorithms in terms of acceptance ratio and revenue to cost ratio.

The rest of this paper is organized as follows. In Section 2, we present the network model and the evaluation indicators of VNE. In Section 3, we give a multi-objective optimization integer linear programming formulation for the VNE problem. In Section 4, we analyze the topology importance of each node in a network, and define the topology potential of a node and the topology potential entropy of the network. In Section 5, we propose our novel algorithm. In Section 6, we perform a broad simulation of the algorithms and discuss the simulation results. Finally, in Section 7, we conclude this paper.

## 2. Network Model and Evaluation Indicators

### 2.1. Physical Network

The physical network can be modeled as a weighted undirected graph $G^{p}=\left(N^{p},E^{p},A_{n}^{p},A_{e}^{p}\right)$, where $N^{p}$ is the set of physical nodes, $E^{p}$ is the set of physical links, $A_{n}^{p}$ is the set of the attributes of physical nodes, and $A_{e}^{p}$ is the set of the attributes of physical links. For a given physical node $n^{p}\in N^{p}$, its attributes include the available CPU (Central Processing Unit) resource and location information, denoted as $cpu\left(n^{p}\right)$ and $loc\left(n^{p}\right)$, respectively. Similarly, for a given physical link $e^{p}\in E^{p}$, its attribute is the available bandwidth, denoted as $b\left(e^{p}\right)$.

### 2.2. Virtual Network Request

Similar to the physical network, the virtual network can also be modeled as a weighted undirected graph $G^{v}=\left(N^{v},E^{v},C_{n}^{v},C_{e}^{v}\right)$, where $N^{v}$ represents the set of virtual nodes, $E^{v}$ represents the set of virtual links, $C_{n}^{v}$ represents the set of the constraint attributes of virtual nodes, and $C_{e}^{v}$ represents the set of the constraint attributes of virtual links. For a given virtual node $n^{v}\in N^{v}$, its attributes include the required CPU resource and location information, denoted as $cpu\left(n^{v}\right)$ and $loc\left(n^{v}\right)$, respectively. For a given virtual link $e^{v}\in E^{v}$, its attribute is the required bandwidth, denoted as $b\left(e^{v}\right)$. Based on the virtual network model above, the k-th arrived VNR can be considered as $VNR_{k}=\left(G^{v},t_{a},t_{e}\right)$, where $G^{v}$ represents the virtual network topology, $t_{a}$ represents the arrive time of the VNR, and $t_{e}$ represents the end time of the VNR.

### 2.3. Evaluation Indicators

The optimization goal of VNE is to efficiently and reasonably utilize the physical network resources in the entire VNE process. In this paper, we use acceptance ratio and revenue to cost ratio as the evaluation indicators for the VNE algorithm.

The VNE acceptance ratio is the ratio of the number of successfully embedded VNRs to the number of total arrived VNRs in a period of time, which can be defined as Formula (1).

(1) $$\eta \left(T\right)=\frac{\sum_{t=0}^{T}\left|VNR_{succ}\right|}{\sum_{t=0}^{T}\left|VNR_{arri}\right|}$$

where $VNR_{succ}$ represents the set of VNRs which have been successfully embedded during the time from 0 to T, and $VNR_{arri}$ represents the set of VNRs which have arrived during the time from 0 to *T*. We can see that the higher the acceptance ratio, the more are VNRs embedded successfully in a certain period of time.

The revenue of accepting $VNR_{k}=\left(G^{v},t_{a},t_{e}\right)$ at time t refers to the total resource it demands, which can be defined as Formula (2).

(2) $$R\left(G^{v},t\right)=\sum_{n^{s}\in N^{v}}cpu\left(n^{s}\right)+\sum_{e^{st}\in E^{v}}b\left(e^{st}\right)$$

The cost of accepting $VNR_{k}=\left(G^{v},t_{a},t_{e}\right)$ at time t is the total physical resource allocated to it, which can be defined as Formula (3).

(3) $$C\left(G^{v},t\right)=\sum_{n^{s}\in N^{v}}\sum_{n^{i}\in N^{p}}x_{i}^{s}\cdot cpu\left(n^{s}\right)+\sum_{e^{st}\in E^{v}}\sum_{e^{ij}\in E^{p}}y_{ij}^{st}\cdot b\left(e^{st}\right)$$

where $x_{i}^{s}$ is a binary variable denoting the embedding relation between the virtual node $n^{s}$ and the physical node $n^{i}$, and $y_{ij}^{st}$ is a binary variable denoting the embedding relation between the virtual link $e^{st}$ and the physical link $e^{ij}$. The definitions of $x_{i}^{s}$ and $y_{ij}^{st}$ are shown in Formulas (4) and (5).

(4) $$x_{i}^{s}=\left\{ \begin{array}{l} 1,\text{ if virtual node }n^{s}\text{ is embedded onto physical node }n^{i}\\ 0,\text{ otherwise} \end{array}\right.$$

(5) $$y_{ij}^{st}=\left\{ \begin{array}{l} 1,\text{ if virtual link }e^{st}\text{ is routed through physical link }e^{ij}\\ 0,\text{ otherwise} \end{array}\right.$$

The revenue to cost ratio is defined as the ratio of the embedding revenue to the embedding cost in a certain period of time, as shown in the Formula (6).

(6) $$\varphi \left(T\right)=\frac{\sum_{t=0}^{T}\sum_{G^{{}_{v}}\in VNR_{succ}}R\left(G^{v},t\right)}{\sum_{t=0}^{T}\sum_{G^{{}_{v}}\in VNR_{succ}}C\left(G^{v},t\right)}$$

where we can see that φ(T) reflects the resource utilization efficiency of the physical network. The larger φ(T), the higher is the utilization efficiency of the physical resource.

## 3. Problem Formulation

### 3.1. Objective Functions

For the online VNE problem, the arrival time, resource requirements, and time of life of the VNRs are unknown. When a VNR arrives, if the embedding objective is only to reduce the embedding cost, more bottleneck links may be generated in the physical network, which will reduce the ability of the physical network to accept subsequent VNRs; if the embedding objective is only to reduce the number of bottleneck links in the physical network, a physical path with more hops may be required to host each embedded virtual link, which will increase the embedding cost of the VNR. Therefore, in this paper, the VNE problem is modeled as a multi-objective integer linear programming model, whose objective functions are to minimize the embedding cost of the VNR as well as the number of bottleneck links in the physical network, such as Formula (7) and Formula (8).

(7) $$\min\sum_{n^{s}\in N^{v}}\sum_{n^{i}\in N^{p}}x_{i}^{s}\cdot cpu\left(n^{s}\right)+\sum_{e^{st}\in E^{v}}\sum_{e^{ij}\in E^{p}}y_{ij}^{st}\cdot b\left(e^{st}\right)$$

(8) $$\min\sum_{e^{p}\in E^{p}}bl\left(e^{p}\right)$$

In Formula (8), $bl\left(e^{p}\right)$ is a binary variable denoting whether $e^{p}$ is a bottleneck link. $bl\left(e^{p}\right)$ is 1 if the physical link $e^{p}$ is a bottleneck link, and 0 otherwise. In this paper, when the utilization of a physical link is more than 80%, we consider it to be a bottleneck link.

### 3.2. Node Constraints

First, the remaining available CPU resource of a physical node $n^{i}$ should not be less than the CPU resource requirement of the virtual node $n^{s}$ which is embedded onto $n^{i}$, such as Formula (9). Second, the Euclidean distance between the physical node and its hosted virtual node should meet the requirements of the virtual node for distance, such as Formula (10). Third, each physical node can only host at the most one virtual node in the same VNR, such as Formula (11). Fourth, each virtual node can only be embedded onto one physical node, such as Formula (12). Finally, the value of $x_{i}^{s}$, which is used to represent the node embedding relationship, should be 0 or 1, such as Formula (13).

(9) $$\forall n^{s}\in N^{v},\forall n^{i}\in N^{p},x_{i}^{s}\cdot cpu\left(n^{s}\right)\leq cpu\left(n^{i}\right)$$

(10) $$\forall n^{s}\in N^{v},\forall n^{i}\in N^{p},x_{i}^{s}\cdot dis\left(loc\left(n^{s}\right),loc\left(n^{i}\right)\right)\leq D\left(n^{s}\right)$$

(11) $$\forall n^{i}\in N^{p},\sum_{n^{s}\in N^{v}}x_{i}^{s}\leq 1$$

(12) $$\forall n^{s}\in N^{v},\sum_{n^{i}\in N^{p}}x_{i}^{s}=1$$

(13) $$\forall n^{s}\in N^{v},\forall n^{i}\in N^{p},x_{i}^{s}\in \left\{0,1\right\}$$

### 3.3. Link Constraints

First, the available bandwidth of a physical link $e^{ij}$ should not be less than the bandwidth requirement of the virtual links which are embedded onto $e^{ij}$, such as Formula (14). Second, it is necessary to consider the connectivity constraints of the link to ensure that every virtual link is embedded onto a valid physical path, such as Formula (15). Finally, the value of $y_{ij}^{st}$, which is used to represent the link embedding relationship, should be 0 or 1, such as Formula (16).

(14) $$\forall e^{ij}\in E^{p},\sum_{e^{st}\in E^{v}}y_{ij}^{st}\cdot b\left(e^{st}\right)\leq b\left(e^{ij}\right)$$

(15) $$\forall n^{i}\in N^{p},\forall e^{st}\in E^{v},\sum_{n^{j}\in N^{p}}y_{ij}^{st}-\sum_{n^{j}\in N^{p}}y_{ji}^{st}=x_{i}^{s}-x_{i}^{t}$$

(16) $$\forall e^{st}\in E^{v},\forall e^{ij}\in E^{p},y_{ij}^{st}\in \left\{0,1\right\}$$

## 4. Node Topology Potential and Network Topology Potential Entropy

At present, there are many typical indicators to evaluate the topology importance of nodes in a network, such as betweenness centrality, degree centrality, and closeness centrality [16,20] etc. However, these indicators either have strong one-sidedness, or emphasize the role of a single node. In physics, the concept of field is used to describe the interaction between two non-contact objects, such as the gravity field and the electromagnetic field. Inspired by the idea above, the field theory was introduced into the abstract data field [22], and the topology potential proposed to assess the importance of the nodes in complex networks [23].

Based on the existing researches, in order to assess the topology importance of each node in a network, we assume that every node in the network creates a virtual field around itself, overlapping with fields of other nodes, so that each node has a different topology potential. Considering that the topology importance of each node is more affected by the performance of its neighboring nodes, we use the Gaussian function that is able to represent the short-range field well to describe the topology potential for each node in the space around it. The topology potential of node $n_{m}$ in a network can be formalized as Formula (17).

(17) $$TP\left(n_{m}\right)=\sum_{k=1}^{\left|N\right|}c\left(n_{k}\right)\cdot \exp\left[-{\left(\frac{dis\left(n_{m},n_{k}\right)}{\sigma }\right)}^{2}\right]$$

where |N| represents the number of nodes in the network; *c*($n_{k}$) represents the topology weight of node *n_k_*; $dis\left(n_{m},n_{k}\right)$ represents the shortest distance between node $n_{m}$ and $n_{k}$; parameter σ is used to control the influence region of each node, called distance factor. Considering that the bandwidth of the outgoing links of a node is larger, its influence on the surrounding nodes is stronger, so the node’s topology weight is defined as Formula (18).

(18) $$c\left(n_{k}\right)=\sum_{e\in E\left(n_{k}\right)}b\left(e\right)$$

where $E\left(n_{k}\right)$ represents the adjacent link set of node $n_{k}$.

According to the property of the Gaussian function, when the value of σ is large, the influence range of every node in the network is larger; when the value of σ is small, the influence range of every node in the network is smaller. In order to select an appropriate value of σ, the topology potential entropy of the network is defined as Formula (19), and the value of σ is obtained by setting the topology potential entropy to be minimum.

(19) $$H_{TP}=-\sum_{m=1}^{\left|N\right|}\frac{TP\left(n_{m}\right)}{\sum_{k=1}^{\left|N\right|}TP\left(n_{k}\right)}\cdot \ln\left(\frac{TP\left(n_{m}\right)}{\sum_{k=1}^{\left|N\right|}TP\left(n_{k}\right)}\right)$$

## 5. VNE-TP Algorithm Design

According to the literature [24,25], the multi-objective integer linear programming model constructed in Section 3 belongs to the NP-hard problem. When the problem size is small, tools such as GLPK and CPLEX [10,11] can be used for an exact solution. When the problem size is large, it is difficult to find the optimal solution within a limited time. To solve this problem, a novel two-stage heuristic algorithm named VNE-TP was designed as in this section.

### 5.1. Node Embedding Algorithm

In the node embedding stage, firstly, the embedding sequence of virtual nodes is constructed according to the topology potential and resource requirement of virtual nodes, and then the virtual nodes are embedded sequentially onto the physical nodes with the best comprehensive ability. The specific steps of the node embedding algorithm are as follows.

Step 1: Calculate the embedding weight of the virtual nodes. Considering that the virtual node with higher topology potential and more resource requirement should be priority embedded because of the limited resource of the physical nodes, the embedding weight of a virtual node is defined as Formula (20).

(20) $$EW\left(n^{v}\right)=TP\left(n^{v}\right)\cdot R\left(n^{v}\right)$$

where $R\left(n^{v}\right)$ represents the resource requirement of virtual node $n^{v}$. The definition of $R\left(n^{v}\right)$ is shown in Formula (21).

(21) $$R\left(n^{v}\right)=cpu\left(n^{v}\right)\cdot \sum_{e^{v}\in E(n^{v})}b\left(e^{v}\right)$$

where $E\left(n^{v}\right)$ represents the adjacent link set of virtual node $n^{v}$.

Step 2: Construct the embedding sequence of virtual nodes. In order to make the virtual nodes near each other in the virtual network remain close to each other after embedding onto the physical nodes, which reduces the embedding cost in the link embedding stage, we construct the virtual node embedding sequence as follows. First, we select the virtual node with maximum embedding weight value as the root node and run the breadth-first search algorithm. Second, we sort the remaining virtual nodes by the distances from themselves to the root node in ascending order. Finally, if there are two virtual nodes with equal distance to the root node, then we sort them by the embedding weight in descending order.

Step 3: Calculate the set of candidate physical nodes for the virtual node to be embedded. For the i-th virtual node $n_{i}^{v}$ in the embedding sequence, according to the node constraints of Formulas ((9)–(13)), select its set of candidate physical nodes, that is $\Omega \left(n_{i}^{v}\right)$.

Step 4: Complete the embedding of the i-th virtual node $n_{i}^{v}$. For $n^{p}\in \Omega \left(n_{i}^{v}\right)$, considering the topology attribute, resource ability attribute, and adjacency relationship, we introduce the node embedding function (NEF) in this paper and embed the virtual node $n_{i}^{v}$ to the physical node whose NEF value is maximum. The definition of NEF is as Formula (22).

(22) $$NEF\left(n^{p}\right)=\frac{TP\left(n^{p}\right)\cdot R\left(n^{p}\right)}{DS\left(n^{p}\right)+\varepsilon }$$

where $R\left(n^{p}\right)$ represents the resource capability of the physical node $n^{p}$, $DS\left(n^{p}\right)$ represents the hops from $n^{p}$ to the physical nodes which have hosted the neighbors of $n_{i}^{v}$, and $\varepsilon =10^{-4}$ is a parameter to prevent the dividend being zero. The definition of $R\left(n^{p}\right)$ is shown in Formula (23).

(23) $$R\left(n^{p}\right)=cpu\left(n^{p}\right)\cdot \sum_{e^{p}\in E\left(n^{p}\right)}b\left(e^{p}\right)$$

where $E\left(n^{p}\right)$ represents the adjacent link set of the physical node $n^{p}$.

The reasons why NEF is selected as the physical node selection metric are mainly due to the following points. First, a physical node with larger $TP\left(n^{p}\right)$ value indicates that its topology potential is higher, and selecting it for embedding the virtual node will facilitate the subsequent virtual link embedding. Second, a physical node with larger $R\left(n^{p}\right)$ value indicates that the node’s available resource is higher, and embedding the virtual node onto it will balance the node stress of the physical network. Finally, a physical node with smaller $DS\left(n^{p}\right)$ value indicates that there are fewer hops between it and the physical nodes that have hosted virtual nodes, and the embedding virtual node onto it will reduce the embedding cost of the subsequent virtual link embedding.

Step 5: Skip to step 3 and continue if the embedding of all virtual nodes is not completed.

The detail pseudo-code of the node embedding algorithm is given by Algorithm 1.

**Algorithm 1** Node Embedding Algorithm of VNE-TP**Input:** Physical network $G^{p}$, virtual network $G^{v}$ **Output:**
*Node_Embedding_List*
**for** every virtual node $n^{v}\in N^{v}$ **do** Calculate $EW\left(n^{v}\right)$**end for**Construct the embedding sequence of virtual nodes by running the breadth-first search algorithm, and record the result into *VirtualNodeList***for**$i=1:\left|N^{v}\right|$**do**  Calculate $\Omega \left(n_{i}^{v}\right)$ for the i-th virtual node in the *VirtualNodeList*  **if** $\Omega \left(n_{i}^{v}\right)\subseteq \emptyset$ **then**   **return** *NODE_EMBEDDING_FAILED*  **else**
   **for** $j=1:\left|\Omega \left(n_{i}^{v}\right)\right|$ **do**    Calculate $NEF\left(n_{j}^{p}\right)$ for the j-th physical node in $\Omega \left(n_{i}^{v}\right)$   **end for**   Embed $n_{i}^{v}$ to $n^{p}$ with the largest NEF value, and record the result into *Node_Embedding_List*  **end if****end for****return***Node_Embedding_List*


### 5.2. Link Embedding Algorithm

In the link embedding stage, the embedding sequence of the virtual links is constructed according to the bandwidth requirement of the virtual links first in descending order, and then the virtual link is embedded onto the physical path with the best comprehensive capability. The specific steps of the link embedding algorithm are as follows.

Step 1: Construct the embedding sequence of the virtual links. According to the bandwidth requirements, the virtual links are arranged in descending order. Thus, it is possible to preferentially embed the virtual links which are difficult to be embedded.

Step 2: Calculate the set of candidate physical paths for the virtual link to be embedded. For the i-th virtual link $e_{i}^{st}$ in the embedding sequence, first delete physical links that do not satisfy bandwidth constraint in the physical network, then select its set of candidate physical paths $\Omega \left(e_{i}^{st}\right)$ by adopting the k-shortest path [26] algorithm.

Step 3: Complete the embedding of the i-th virtual link $e_{i}^{st}$. Considering that the virtual link embedding should be helpful for coordinating the physical link resource consumption and reducing the embedding cost, we introduce the path embedding function (PEF) in this paper and embed the virtual link $e_{i}^{st}$ to the physical path whose PEF value is maximum. The definition of PEF is as Formula (24).

(24) $$PEF\left(p\right)=\frac{b\left(p\right)}{b\left(e_{i}^{st}\right)}\cdot \frac{1}{hops\left(p\right)}$$

where b(p) represents the available bandwidth of the physical path p, and hops(p) represents the hops of the physical path p.

The reasons why PEF is selected as the physical path selection metric are as follows. First, the physical path with larger b(p) value indicates that the path’s available resource is higher, and the embedding virtual link onto it will balance the link stress of the physical network, thereby reducing the number of bottleneck links in the physical network. Second, the physical path with smaller hops(p) value indicates that the path has fewer hops, and the embedding virtual link onto it will reduce the embedding cost.

Step 4: Skip to step 2 and continue if the embedding of all virtual links is not completed.

The detail pseudo-code of the link embedding algorithm is given by Algorithm 2.

**Algorithm 2** Link Embedding Algorithm of VNE-TP**Input:** Physical network $G^{p}$, virtual network $G^{v}$, *Node_Embedding_List* **Output:**
*Link_Embedding_List*
Construct the embedding sequence of virtual links, and record the result into *VirtualLinkList***for**$i=1:\left|E^{v}\right|$**do**  Calculate $\Omega \left(e_{i}^{st}\right)$ for the i-th virtual link in the *VirtualLinkList*  **if** $\Omega \left(e_{i}^{st}\right)\subseteq \emptyset$ **then**   **return** *LINK_EMBEDDING_FAILED*  **else**
   **for** $j=1:\left|\Omega \left(e_{i}^{st}\right)\right|$ **do**    Calculate $PEF\left(p_{j}\right)$ for the j-th physical path in $\Omega \left(n_{i}^{v}\right)$   **end for**   Embed $e_{i}^{st}$ to p with the largest PEF value, and record the result into *Link_Embedding_List*  **end if****end for****return***Link_Embedding_List*


## 6. Performance Evaluation

### 6.1. Simulation Environments

#### 6.1.1. Network Settings

In this paper, similar to literature of ref. [20], we use the GT-ITM tool [27] to generate the topologies of the physical and virtual networks. The physical network is set to have 100 nodes and 500 links, and the positions of the physical nodes follow a random distribution in a scope of 1000×1000. The initial CPU resource and bandwidth resource of the physical nodes and links are uniformly distributed between 50 and 100. 

For each VNR, the number of virtual nodes is uniformly distributed between four and eight, and the average link connectivity rate is set to be 50%. The positions of the virtual nodes follow a random distribution in the scope of 1000 × 1000. The CPU resource requirement and bandwidth resource requirement of virtual nodes and links are uniformly distributed between 1 and 50. The arrival rate of VNRs follows a Poisson process, and the lifetime of each VNR follows an exponential distribution with an average of 500 time units. The total time of every simulation is 30,000 time units. In order to reduce the influence of random factors on the experimental results, each simulation is carried out 10 times, and the average values are taken as the final results.

#### 6.1.2. Comparison Methods

In this paper, we set the position constraint to be $D\left(n^{s}\right)=400$ and compared three algorithms that are listed in Table 1. VNE-TP is our proposed algorithm, VNE-TOPSIS and VNE-MCRR are the algorithms proposed in the literature [20] and [19] respectively.

### 6.2. Evaluation Results

#### 6.2.1. Performance Comparison

**Figure 1:**
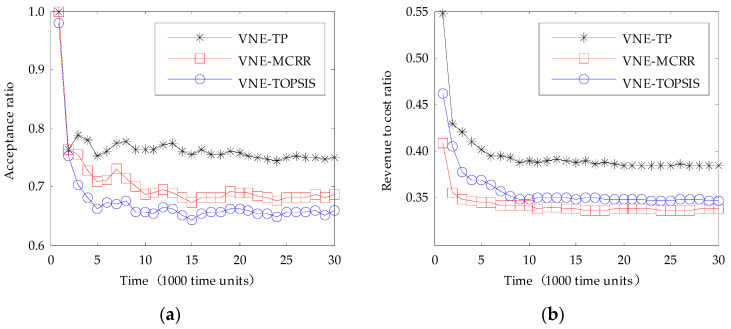
Comparison between our algorithm and the existing algorithms: (**a**) Acceptance ratio over time; (**b**) Revenue to cost ratio over time. VNE-TP is our proposed algorithm, VNE-TOPSIS and VNE-MCRR are the algorithms proposed in the literature [20] and [19] respectively.

When the arrival rate of VNRs follows a Poisson process with a mean arrival rate of 0.06 VNR per each time unit, the comparison results of the three algorithms are shown in Figure 1.

As shown in Figure 1a, the VNE-TP algorithm has the highest acceptance ratio during the entire simulation time. In the initial time period, the acceptance ratio of the three algorithms is relatively high, because the available resources of the physical network are initially abundant. With increasing simulation time and the arriving of the VNRs, the available resources of the physical network gradually decrease, and the acceptance ratio of the three algorithms decreases gradually and tends to be stable after about 10,000 time units. When the simulation time reaches 30,000 time units, the acceptance ratio of the VNE-TP algorithm is 75.06%, which is about 9.4% and 13.9% higher than that of VNE-MCRR and VNE-TOPSIS algorithms respectively. Compared with other algorithms, the VNE-TP algorithm takes more consideration of the topology attributes of the nodes in the node embedding stage, and takes a comprehensive consideration of the bandwidth and hops of the path in the link embedding stage. These measures make the node embedding more reasonable and the link embedding more optimized, so the acceptance ratio is increased.

As can be seen from Figure 1b, the VNE-TP algorithm has the highest revenue to cost ratio during the entire simulation time. In the initial stage of the simulation, with the increase of time, the revenue to cost ratio of the three algorithms is decreased. The reason is that the available resources of the physical network is decreased with the increased number of arrived VNRs, and the virtual links have to be embedded onto the physical paths with more hops, which make the embedding cost increase. When the simulation time is longer than 10,000 time units, the arrival rate and leave rate of VNRs tend to be the same, so the available resources of the physical network tend to be stable, and the revenue to cost ratio also tends to be stable. In the stable situation, the revenue to cost ratio of VNE-TP algorithm is about 38.54%, which is about 14.1% and 10.9% higher than that of VNE-MCRR and VNE-TOPSIS algorithms respectively. 

#### 6.2.2. Evaluation with Different Arrival Rate of VNRs

**Figure 2:**
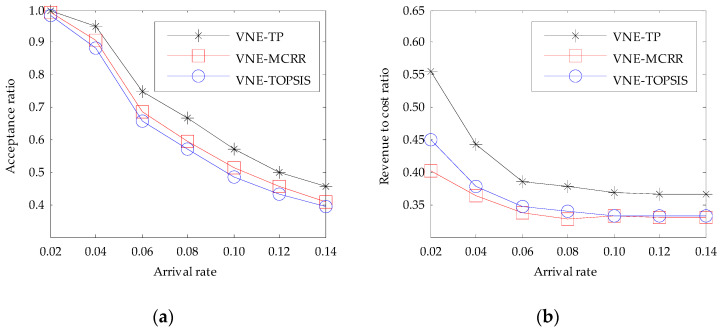
The influence of arrival rate of virtual network requests (VNRs): (**a**) Acceptance ratio with arrival rate; (**b**) Revenue to cost ratio with arrival rate.

In order to further verify the performance of the proposed algorithm, we investigated the influence of the arrival rate of VNRs on the performance of it. When the arrival rate of VNRs follows a Poisson process with a mean arrival rate of 0.02, 0.04, 0.06, 0.08, 0.10, 0.12, and 0.14 VNR per each time unit, respectively, the performance of the three algorithms compared in this paper can be seen in Figure 2.

From Figure 2a, we can see that the VNE-TP algorithm has the highest acceptance ratio in all the arrival rates of VNRs. For example, when the arrival rate is 0.14 VNR per each time unit, the acceptance ratio of VNE-TP algorithm is about 45.39%, which is about 11.4% and 14.9% higher than that of VNE-MCRR and VNE-TOPSIS algorithms respectively. In addition, we can see that the acceptance ratio of the three algorithms is decreased with the increase of the arrival rate of VNRs. This is because the higher arrival rate of VNRs, the larger amount of arrived VNRs within the same time, and the available resources of the physical network are limited. All of these will result in a large amount of VNRs which cannot be embedded successfully.

From Figure 2b, we can see that the VNE-TP algorithm has the highest revenue to cost ratio in all the arrival rates of VNRs. For example, when the arrival rate is 0.14 VNR per each time unit, the revenue to cost ratio of VNE-TP algorithm is about 36.49%, which is about 10.2% and 10.0% higher than that of VNE-MCRR and VNE-TOPSIS algorithms respectively. When the arrival rate of VNRs is less than 0.10 VNR per each time unit, the revenue to cost ratio of the three algorithms decreases with the increase of the arrival rate of VNRs. However, when the arrival rate of VNRs is more than 0.10 VNR per each time unit, the revenue to cost ratio of the three algorithms tends to be stable. The reasons for this result are as follows:

(1) As the arrival rate of VNRs increases, the number of arrived VNRs increases. In order to accept as many VNRs as possible, the virtual links have to be embedded onto the physical paths with more hops, which make the revenue to cost ratio decrease.
(2) When the arrival rate of VNRs increases to a certain extent, the number of VNRs that the physical network can host no longer increases as the arrival rate of VNRs increases. Therefore, the revenue to cost ratio tends to be stable.

#### 6.2.3. Evaluation with Different Access Control Conditions

**Figure 3:**
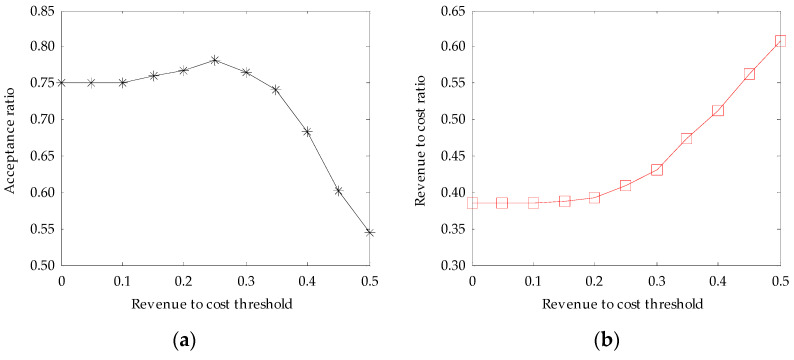
The influence of access control conditions: (**a**) Acceptance ratio with threshold; (**b**) Revenue to cost ratio with threshold.

The VNE-TP algorithm has greedy characteristics in nature, which accepts all the VNRs that satisfy the constraints. If the revenue to cost ratio of a VNR is too low, it will take up more physical resources, thereby affecting the acceptance ratio and the revenue to cost ratio of subsequent VNRs. Therefore, in order to further optimize the performance of VNE-TP algorithm, in this paper we studied the impact of access control conditions on the performance of the VNE-TP algorithm by considering the revenue to cost thresholds as the access control conditions. The strategy of the access control is to proactively reject the VNRs whose revenue to cost ratios are lower than the threshold. The simulation results are listed in Figure 3.

From Figure 3a, we can see that the acceptance ratio of the VNE-TP algorithm increases a little with the increase of the revenue to cost threshold first and then decreases rapidly. When the revenue to cost threshold is 0.25, the VNE-TP algorithm has the highest acceptance ratio of about 78.15%, which is about 4.1% higher than that of the VNE-TP algorithm without considering the access control.

From Figure 3b, we can see that the revenue to cost ratio of the VNE-TP algorithm increases with the increase of the revenue to cost threshold. When the revenue to cost threshold is 0.25, the VNE-TP algorithm has a revenue to cost ratio of about 40.94%, which is about 6.2% higher than that of the VNE-TP algorithm without considering the access control.

## 7. Conclusions

In this paper, the VNE problem was investigated by introducing field theory in physics. A multi-objective integer linear programming model for VNE was constructed and a two-stage coordinated heuristic VNE algorithm, named VNE-TP, proposed. Simulation results show that the acceptance ratio and the revenue to cost ratio of the VNE-TP algorithm are about 10% higher than the existing VNE algorithms in all of the simulation conditions. The influence of the access control condition on the performance of VNE-TP algorithm was analyzed, and it was concluded that by selecting an appropriate revenue to cost threshold, the acceptance ratio and the revenue to cost ratio of the VNE-TP algorithm can be further improved.

However, we did not consider the reliability of the physical network in this paper. In the real world, the nodes and links of the physical network may fail during the working process. Therefore, in the next step, focusing on the sudden failure of the physical network, we will introduce reliability into the VNE process, and study the reliable VNE algorithm.

## Figures and Tables

**Table 1:** Algorithm comparison.

Notation	Description
VNE-TP	In the node embedding stage, the topology potential, the resource capability and the distance attribute are considered to rank the nodes. In the link embedding stage, the available bandwidth and the hops of the paths are considered, and a k-shortest path algorithm is used.
VNE-TOPSIS	In the node embedding stage, five novel node attributes are proposed, and the nodes are ranked based on TOPSIS. In the link embedding stage, a shortest-path based algorithm is used.
VNE-MCRR	In the node embedding stage, the Markov Reward Model is introduced, and the nodes are ranked based on Markov Reward Processes. In the link embedding stage, a shortest-path based algorithm is used.
